# Prevalence of fibrodysplasia ossificans progressiva (FOP) in the United States: estimate from three treatment centers and a patient organization

**DOI:** 10.1186/s13023-021-01983-2

**Published:** 2021-08-05

**Authors:** Robert J. Pignolo, Edward C. Hsiao, Genevieve Baujat, David Lapidus, Adam Sherman, Frederick S. Kaplan

**Affiliations:** 1 Geriatric Medicine & Gerontology, Robert and Arlene Kogod Professor of Geriatric Medicine, Mayo Clinic College of Medicine, Mayo Clinic, Rochester, MN USA; 2 Robert L. Kroc Chair in Rheumatic and Connective Tissue Diseases III, Division of Endocrinology and Metabolism, University of California, San Francisco, CA USA; 3 Department of Medicine, Institute for Human Genetics, University of California, San Francisco, CA USA; 4 Program in Craniofacial Biology, University of California, San Francisco, CA USA; 5 Department of Clinical Genetics, INSERM U1163, Paris-Descartes University, Imagine Institute, Necker-Enfants Malades Hospital, Paris, France; 6 LapidusData Inc., Oklahoma City, OK USA; 7 The International FOP Association, North Kansas City, MO USA; 8 Departments of Orthopaedic Surgery and Medicine, The Perelman School of Medicine, University of Pennsylvania, Philadelphia, PA USA; 9 The Center for Research in FOP & Related Disorders, The Perelman School of Medicine, University of Pennsylvania, Philadelphia, PA USA

**Keywords:** Fibrodysplasia ossificans progressiva, FOP, ACVR1, Heterotopic ossification, Rare disease, Epidemiology, Prevalence

## Abstract

**Background:**

Fibrodysplasia ossificans progressiva (FOP), an ultra-rare, progressive, and permanently disabling disorder of extraskeletal ossification, is characterized by episodic and painful flare-ups and irreversible heterotopic ossification in muscles, tendons, and ligaments. Prevalence estimates have been hindered by the rarity of FOP and the heterogeneity of disease presentation. This study aimed to provide a baseline prevalence of FOP in the United States, based on contact with one of 3 leading treatment centers for FOP (University of Pennsylvania, Mayo Clinic, or University of California San Francisco), the International Fibrodysplasia Ossificans Progressiva Association (IFOPA) membership list, or the IFOPA FOP Registry through July 22, 2020.

**Results:**

Patient records were reviewed, collected, and deduplicated using first and last name initials, sex, state, and year of birth. A Kaplan–Meier survival curve was applied to each individual patient to estimate the probability that he or she was still alive, and a probability-weighted net prevalence estimate was calculated. After deduplication, 373 unique patients were identified in the United States, 294 of whom who were not listed as deceased in any list. The average time since last contact for 284 patients was 1.5 years. Based on the application of the survival probability, it is estimated that 279 of these patients were alive on the prevalence date (22 July 2020). An adjusted prevalence of 0.88 per million US residents was calculated using either an average survival rate estimate of 98.4% or a conservative survival rate estimate of 92.3% (based on the Kaplan–Meier survival curve from a previous study) and the US Census 2020 estimate of 329,992,681 on prevalence day.

**Conclusions:**

This study suggests that the prevalence of FOP is higher than the often-cited value of 0.5 per million. Even so, because inclusion in this study was contingent upon treatment by the authors, IFOPA membership with confirmed clinical diagnosis, and the FOP Registry, the prevalence of FOP in the US may be higher than that identified here. Thus, it is imperative that efforts be made to identify and provide expert care for patients with this ultra-rare, significantly debilitating disease.

## Introduction

Fibrodysplasia ossificans progressiva (FOP; OMIM #135100) is an ultra-rare, progressive, and permanently disabling disorder of extraskeletal ossification. FOP is characterized by episodic flare-ups and irreversible heterotopic ossification (HO) in muscles, tendons, and ligaments. This often results in a permanent loss of mobility, decreased quality of life, and a shortened lifespan [1–3]. FOP is often misdiagnosed, and early diagnosis of FOP is important to minimize the risk of irreversible harm from unnecessary invasive testing, intramuscular injections, and other tissue trauma [4].

Two hallmark clinical features define classic FOP: malformation of the great toes and progressive HO. Other skeletal features observed in patients with FOP include shortened thumbs, cervical spine malformations, short broad femoral necks, and distal femora and proximal medial tibial osteochondromas [5].

Starting in early childhood, patients with FOP develop episodic, painful inflammatory flare-ups involving soft tissues, such as aponeuroses, fascia, ligaments, tendons, and skeletal muscle. Swelling and pain are the most consistent early findings of flare-ups, and other common major symptoms may include stiffness, warmth, and decreased movement, as well as changes in mood or behavior, fever, loss of appetite, and lethargy [6]. Although some flare-ups spontaneously resolve, more often they lead to HO of soft connective tissues and permanent immobility [4, 7, 8]. Flare-ups may be caused by inflammation, influenza-like illnesses, mandibular blocks for dental work, intramuscular immunizations, muscle fatigue, and blunt muscle trauma from bumps, bruises, and falls [7, 9]. Care for patients with FOP requires careful medical planning to reduce injuries [10]. There are currently no directed medical treatments for FOP.

HO in FOP occurs through an endochondral pathway [11, 12]. Although most cases occur de novo from a spontaneous new mutation, genetic transmission may also occur either maternally or paternally in an autosomal dominant manner [1]. Classic FOP is caused by missense mutations in *ACVR1*, a gene encoding the bone morphogenetic protein (BMP)-type 1 receptor ALK2 [1, 5, 7, 11, 12]. Approximately 97% of identified patients with classic FOP have the same heterozygous, single-nucleotide change in *ACVR1*: 617G > A; R206H [5, 12, 13]. The ACVR1^R206H^ mutation is thought to contribute to leaky activity of ALK2, ultimately resulting in increased BMP pathway signaling and expression of bone-forming genes [14–18]. Activin A promotes increased BMP pathway signaling in FOP through neo-signaling activity by the mutant ACVR1 [17, 19, 20]. The altered gene expression profile leads to activation of mature fibroadipogenic cells, which can form cartilage that is then replaced by heterotopic bone [21].

Although the HO occurs episodically, disability in FOP is cumulative, and most patients are immobilized and confined to a wheelchair by the third decade of life. Individuals with FOP typically require lifelong assistance to perform activities of daily living [4, 7, 8]. While FOP progresses most significantly as a consequence of flare-ups, nearly half of patients with FOP have reported progressive mobility restriction without discrete flare-ups, which suggests possible progressive subclinical HO and/or early progressive degenerative arthropathy [22, 23].

A 2010 study of mortality records from the International FOP Association (IFOPA) from its inception in 1988 through 2006 and the International FOP Clinic at the University of Pennsylvania from 1973 through 2006 found that the median age at time of death for FOP patients was 40 years (range of 3 to 77 years) [2]. When including data from living individuals (as of January 2006) in the IFOPA membership list, the median estimated lifespan was found to be 56 years (95% confidence interval 51 to 60 years). The most common causes of death in patients with FOP included cardiorespiratory failure from thoracic insufficiency syndrome (54%, median age of 42 years) and pneumonia (15%, median age of 40 years) [2].

Estimates of the true prevalence of FOP are evolving, with recent studies suggesting a prevalence higher than the 0.5 per million referenced previously. Attempts to determine prevalence are hampered by the ultra-rarity of FOP and heterogeneity of disease. However, it is notable that prevalence is not believed to differ based on sex, race, or ethnicity [24, 25].

The reported global prevalence of FOP based on patient organization databases varies significantly throughout the world and likely represents an underestimate of the true biological prevalence of FOP. The apparent prevalence of registered and confirmed FOP patients varied substantially from approximately 0.65 per million in North America and 0.47 per million in Western Europe to approximately 0.27 per million in Latin America, 0.05 per million in Africa, to nearly 0.04 per million in the Asia–Pacific region [26]. A study by Connor and Evans published in 1982 identified 44 patients with FOP and estimated a prevalence of 0.61 per million inhabitants in the United Kingdom. This study utilized multimethod case ascertainment, with a focus on a survey of relevant physician specialties [27].

A study by Baujat et al. published in 2017 identified 89 patients with FOP on the date January 1, 2012, determining a prevalence of 1.36 (95% confidence interval 1.1 to 1.7) per million inhabitants in France using a capture-recapture methodology—more than double that of the 1982 study [24, 27]. This study leveraged France’s referral network for rare bone diseases, which concentrates patients at a small number of regional and national centers of excellence, as well as utilizing diagnostic codes from the country’s national health insurance system. Because of the referral system, as well as the severity and unique nature of FOP symptoms, this study is likely to have captured nearly all diagnosed cases. Although false positives may be included and undiagnosed patients could be missing from this estimate, France’s national health insurance system affords universal access, and the number of undiagnosed cases is thought to be low. Therefore, the prevalence reported in the Baujat study is presumed close to, but still less than, the true biological prevalence of FOP in France.

Unlike France, there is no national referral network in the United States, so the methodology of Baujat et al. cannot be duplicated. However, FOP care tends to be centralized in the United States, with most patients being referred to a relatively small number of reference centers. Furthermore, there are few physician experts in FOP, increasing the centralization of care. The IFOPA is an international patient organization, in which people with FOP may enroll to receive education and support services. The IFOPA also manages the FOP Registry, which is an observational, longitudinal study that captures demographic and disease information from people living with FOP [28].

The present study aimed to determine the incidence of FOP in the US by compiling records of patients seen by the authors (University of Pennsylvania, Mayo Clinic, and University of California San Francisco), and those who participated in either the IFOPA membership or the IFOPA Registry.

## Methods

### Study centers and data collection

The study centers included 3 major clinical sites represented by experts in FOP (FSK- University of Pennsylvania; RJP—Mayo Clinic; and ECH—University of California San Francisco) and the IFOPA. The clinicians reviewed patient records, including formal medical records and any other records or notes from patient consultations. The IFOPA supplied data from its FOP Registry as well as its broader membership list. For each FOP patient, the centers each provided as many of the following data fields as possible: initials, year of birth, sex, location of residence, and date of last contact (or the last date when patient was known to be alive), following HIPPA and local Institutional Review Board regulations.

Each center included any patients known to be deceased so that such patients could be eliminated from other lists that may have included them. Likewise, participants also included patients for whom a diagnosis of FOP was considered but later rejected based on clinical or genetic criteria. Only patients residing in the United States were included in this study.

Patient records from the 3 centers contained clinical and/or genetic confirmation of FOP diagnosis. Any form of FOP was accepted for this study, including individuals with both the classical FOP ACVR1^R206H^ mutation as well as the “variant” or non-classical forms of the mutation. Because the IFOPA membership list is self-reported, confirmed diagnosis is not required for membership. Patients whose primary residence was not in the United States were excluded. To ensure that patients who were identified exclusively through IFOPA membership were indeed FOP patients, the IFOPA contacted each of these patients to confirm that they had received a clinical diagnosis and were still living. Of the 34 patients who were contacted, 15 could not be confirmed as having FOP, and were removed from the analysis. Inclusion in the FOP Registry requires genetic test results, clinical diagnosis, or association with a FOP patient group. Submissions were accepted through 22 July 2020, which was the study’s prevalence cutoff date for inclusion in the analysis.

### Data processing

Participants sent their lists to an outside consultant (DL) who deduplicated the lists. A conservative approach was used to avoid overestimating the identified population. Patients from the different lists were merged into a deduplicated identity for each case where no conflicts existed among the fields. At minimum, initials, gender, and year of birth were required to generate a patient identity. Records lacking these data were excluded from the analysis. For patients identified though IFOPA membership alone, the IFOPA confirmed that the patient had received a genetic or clinical diagnosis of FOP. Those patients who could not be contacted or had not received a diagnosis were removed from the analysis.

These data were sufficient to generate a deduplicated list of patients who were known to be alive at a specific point in time (last contact with any of the participants). The dates of last contact ranged from as recent as July 2020 to as long ago as 1990. Some patients may have died in the intervening time since their last contact, so a Kaplan–Meier survival curve was applied to each individual patient to estimate the probability that he or she was still alive on the prevalence date. The survival curve was based on an earlier study (Kaplan et al., 2010) [2] for which the primary investigator was also a participant in the current study (FSK). A multiterm logistical regression (Microsoft Excel) was applied to data from Kaplan et al. [2] to generate a smoothed survival curve with survival probabilities for every age-year. The sum of these survival probabilities for each patient was used to calculate a probability-weighted net prevalence estimate.

The US Census 2020 estimate of 329,992,681 on the prevalence day was used as reference [29].

A date of last contact could not be confirmed for 10 patients, so the survival curve could not be directly applied to them. To generate a conservative (low) estimate of the share of these patients who were still alive on the prevalence day, a survival probability was calculated using the bottom quintile of patients for whom the date of last contact was available. This group of patients had the highest estimated mortality rate (7.7%). This mortality rate was applied to the 10 patients with missing data, resulting in an estimate that approximately 9 of the 10 survived to the prevalence day.

## Results

**Fig. 1:**
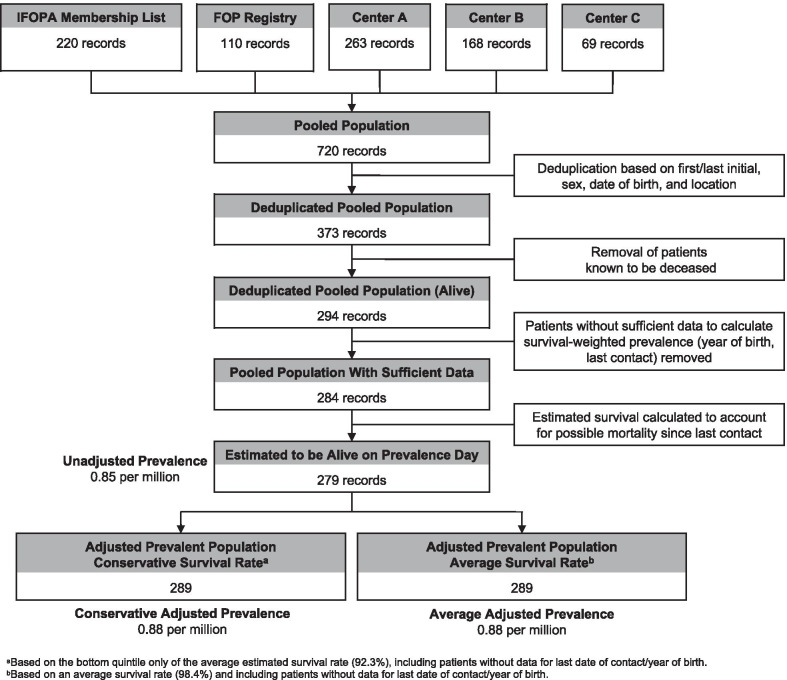
Waterfall of United States FOP patient population estimate on 22 July 2020. A pooled total of 720 patients were identified based on the 3 sources (the IFOPA membership list and FOP Registry were considered together as one source). Following deduplication based on first/last initial, sex, date of birth, and location, 373 unique patients were identified. Of these patients, 294 had not been reported to be deceased. Ten patients without sufficient data (year of birth, date of last contact) to calculate the survival-weighted prevalence were removed. After application of a survival prediction based on patient age at last contact and time since last contact (prevalence date of 22 July 2020), an unadjusted prevalence of 0.85 per million was calculated. After adjusting using a conservative survival rate (92.3%) based on the bottom quintile of the average estimated survival rate reported by Kaplan 2010 [2], an adjusted prevalence of 0.88 per million was calculated. When instead adjusting using the average survival rate of 98.4% reported by Kaplan 2010 [2], the adjusted prevalence is 0.88 per million

A total of 220 patients were identified from the IFOPA membership list, which included 110 patients who were also part of the FOP Registry, and 263, 168, and 69 who were identified from the patient lists of the 3 reference centers, resulting in a pooled collection of 720 records. After deduplication based on first/last initial, sex, year of birth, and location, 373 unique patients were identified in the United States; 294 of these patients were not reported as deceased in any list (Fig. 1).

**Fig. 2:**
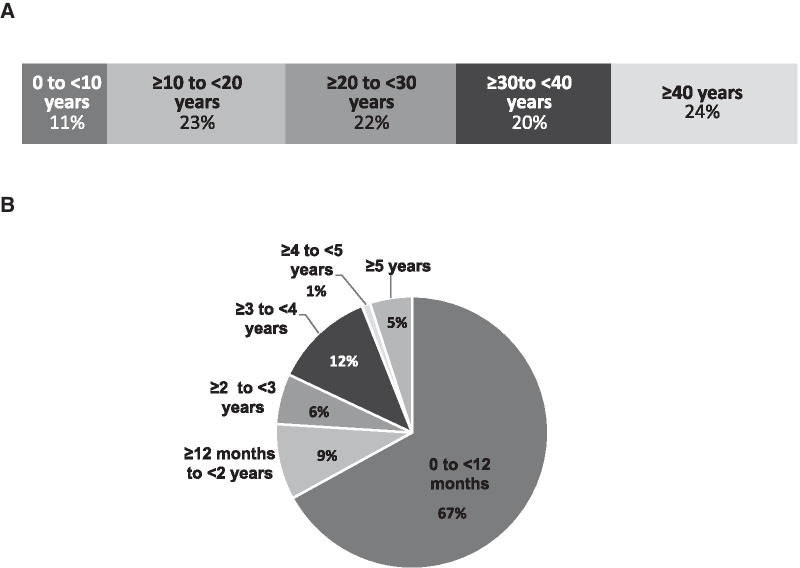
Overview of Patient Demographics. **A** Age distribution of 285 subjects with age data. **B** Time since last contact of 285 subjects with time since last contact data

Of 284 patients with age data, the average age was 29.3 years; 31 (11%) were 0 to < 10 years, 65 (23%) were ≥ 10 to < 20 years, 63 (22%) were ≥ 20 to < 30 years, 58 (20%) were ≥ 30 to < 40 years, and 67 (24%) were ≥ 40 years. The mean time since last contact date from any source based on 284 patients with this information was 1.5 years (median 103 days, range 9 days to 26 years). Of these patients, 190 (67%) had last contact within 0 and < 12 months, 26 (9%) ≥ 12 months to < 2 years, 18 (6%) ≥ 2 years to < 3 years, 33 (12%) ≥ 3 years and < 4 years, 2 (1%) ≥ 4 years and < 5 years, and 15 (5%) ≥ 5 years (Fig. 2).

**Fig. 3:**
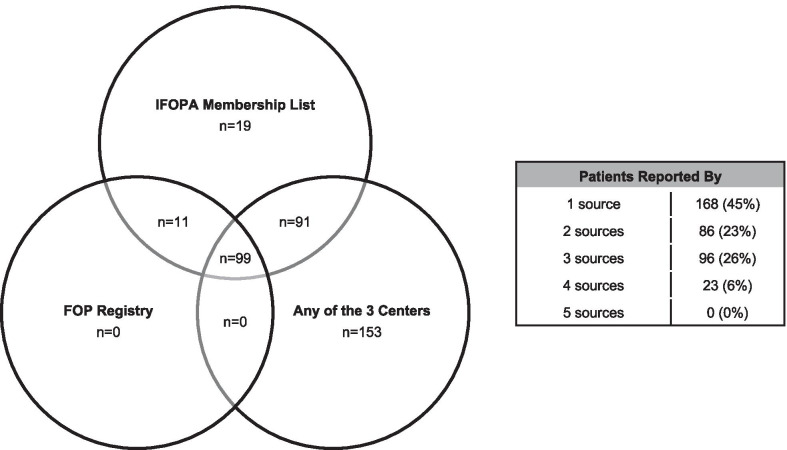
Patient identification overlap. The Venn diagram shows the number of the 373 unique patients identified based on source (IFOPA membership list, FOP Registry, and any of the 3 centers). The term “source” refers to the IFOPA membership list, the FOP Registry, and each of the 3 centers represented by FOP expert clinicians

Figure 3 shows the overlap of patients identified from each source on the Venn diagram. Of the 373 unique patients, 99 (26.5%) were identified by the FOP Registry, the IFOPA membership list, and at least 1 clinician, 91 (24.4%) were identified by the IFOPA membership list and at least 1 clinician, 11 (2.9%) were identified by both the IFOPA membership list and FOP Registry, 153 (41.0%) were identified by 1 or more clinicians, and 19 (5.1%) were identified by only the IFOPA membership list and confirmed by IFOPA staff as having received a clinical diagnosis. When considering the FOP Registry, IFOPA membership, and each clinician as a separate source, 168 (45%) patients were identified by only 1 source, 86 (23%) by 2 sources, 96 (26%) by 3 sources, and 23 (6%) by 4 sources. No patients were identified by all 5 sources. Notably, 5.1% of the total identified patients were unique to the IFOPA’s membership list.

Given that approximately one-third of patients included in the list had not been in contact with a referral site for more than 1 year, it was possible that they were deceased and therefore should be excluded from the prevalence estimates. In order to account for potential deceased patients, the analysis applied the likelihood of survival given patient’s age and date of last contact. Considering these factors, 279 patients were estimated to be alive on the prevalence date in the United States (excluding the 10 patients without sufficient data), which provides an unadjusted prevalence of 0.85 per million. Assuming a conservative survival rate of 92.3% calculated using the bottom quintile only of the average estimated survival rate from Kaplan et al. [2] for patients without data for the last date of contact and/or year of birth, 289 patients were estimated to be alive on the prevalence date, and a conservative adjusted prevalence of 0.88 per million was calculated. Assuming the average survival rate (98.4%, published by Kaplan 2010 [2],) for patients without data for the last date of contact and/or year of birth, the average adjusted prevalence was 0.88 per million (Fig. 1). Both the 98.4% and 92.3% survival rates resulted in the same adjusted prevalence of 0.88 per million because when these rates were applied to the 10 patients without sufficient data to calculate estimated survival, the number of individuals estimated to be alive was 9.8 and 9.2, respectively. These values were added to the number of patients with sufficient data who were estimated to be alive on the prevalence date (279.33), yielding 288.56 and 289.17 individuals. Both of these numbers were rounded to 289 prior to the final calculation of prevalence of 0.88 per million.

## Discussion

This study estimates a prevalence of FOP of 0.88 per million in the United States, based on the 289 unique patients reported by FOP clinical centers, IFOPA Registry, and the IFOPA estimated to be alive on the study’s prevalence day [29]. However, it is highly likely that patients exist outside of this pooled dataset (e.g., patients who have not yet been diagnosed, patients who receive care through physicians who have not connected to the authors’ institutions, or patients who have not connected with the IFOPA). This suggests that the incidence of 0.88 per million calculated here is likely an underestimate.

Notably, our estimate is somewhat higher than those of Connor et al. (1982) of 0.61 per million in the United Kingdom, and that of Liljesthröm et al. (2020) of 0.658 per million in 2016 in North America [26, 27]. It is generally assumed that there is no predilection for sex, race, or ethnicity [6, 27]; however, this assumption has not been formally examined in enough populations to be certain. It is possible that there are unidentified genetic modifiers and/or cultural and environmental factors that would affect prevalence calculations of FOP. In France, the current estimate of prevalence based on the study of Baujat et al. is higher, at 1.36 per million [24]. This French study leveraged a more-centralized system of FOP patient care, so case ascertainment may account for at least part of the difference.

It is important that patients with complex, rare conditions such as FOP have medical input from clinicians with deep experience and expertise in the target disease. While this study shows that there has been some success in achieving patient care among qualified experts in the United States, work remains in order to ensure that patients are connected to national and local care networks. Of the 373 identified patients (some of whom are deceased), 220 (59%) had made contact with the IFOPA, including 19 (5.1%) unique to the IFOPA membership list. There were no patients included in the analysis who were unique to only the FOP Registry. It is important to note that a formal diagnosis of FOP is not required for inclusion in the IFOPA membership list. For this study, patients identified solely though this membership were included in the study only if the IFOPA was able to obtain confirmation from the patient that they had received either a clinical or genetic diagnosis. It is possible that at least some of the 15 IFOPA members with whom the IFOPA could not connect are indeed individuals with FOP: if these 15 individuals are included in the calculation of incidence (applying the most conservative survival rate), the prevalence increases from 0.88 per million to 0.94 per million.

There are some limitations to our study. Data were incomplete for some patients: 10 patients were missing the date of last contact and/or year of birth and thus could not be directly included in the survival probability–weighted prevalence. Further, the survival-weighted prevalence is a theoretical number based on a survival curve published in 2010 including 371 living individuals and 60 reported deaths in FOP patients during a 33-year period (1973 to 2006). That study established the survival curve based on mortality records from the IFOPA (from its inception in 1988 through 2006) as well as the International FOP Clinic at the University of Pennsylvania (from 1973 through 2006) and the membership records of the IFOPA of living individuals in January 2006, and the 1980 US Census. The impact of changes in medical care since this time on survival is unknown.

Other important limitations are that some patients are likely receiving care outside the 3 FOP specialty centers included in this study, are not participating in the FOP Registry, have not joined the IFOPA (or were not able to be reached at the time of this study to confirm diagnosis), have been misdiagnosed, or have not yet received a definitive diagnosis. Together with the conservative methods applied to the calculation of incidence, these factors make it likely that 0.88 per million is an underestimate. According to the FOP Registry, 53.5% of patients had received a misdiagnosis prior to correct diagnosis. In addition, patients are not diagnosed until an average at of 8.3 years [30], which may lead to underestimation of FOP incidence in patients 0 to < 10 years of age. In this study, 11% (n = 31) of patients were 0 to < 10 years, compared to 23% (n = 65) in the ≥ 10 to < 20 years group, and 24% (n = 67) in the ≥ 40 years group. Given the nature of FOP, one would expect the number of patients in this youngest bracket to be at least equal to the older age bracket with the highest number of patients. Therefore, it is likely that a number of patients (both diagnosed and undiagnosed) were not ascertained in this study, and thus not included in the prevalence estimate. This finding is of particular importance in that lack of early diagnosis could lead to absence of injury reduction measures for the patient and/or unnecessary invasive procedures like biopsy or other medical interventions that could stimulate post-traumatic HO.

## Conclusions

This study aimed to determine the prevalence of FOP in the United States and succeeded in estimating the number of FOP patients with access to the expert care and/or association with a patient organization. Taking into account the limitations described above, we report an estimated minimum prevalence of 0.88 per million individuals (1 in 1.14 million people) living in the United States. Our results provide a minimum US prevalence, and also shed light on the importance of allocating resources to ensure a timely diagnosis for FOP patients and building a clinical network which encourages specialized patient care and management.

## Data Availability

The datasets used and/or analysed during the current study are available from the corresponding author on reasonable request.

## References

[1] Pignolo RJ, Shore EM, Kaplan FS (2011). Fibrodysplasia ossificans progressiva: clinical and genetic aspects. Orphanet J Rare Dis.

[2] Kaplan FS, Zasloff MA, Kitterman JA, Shore EM, Hong CC, Rocke DM (2010). Early mortality and cardiorespiratory failure in patients with fibrodysplasia ossificans progressiva. J Bone Joint Surg Am.

[3] Ortiz-Agapito F, Colmenares-Bonilla D (2015). Quality of life of patients with fibrodysplasia ossificans progressiva. J Child Orthop.

[4] Kitterman JA, Kantanie S, Rocke DM, Kaplan FS (2005). Iatrogenic harm caused by diagnostic errors in fibrodysplasia ossificans progressiva. Pediatrics.

[5] Kaplan FS, Xu M, Seemann P (2009). Classic and atypical fibrodysplasia ossificans progressiva (FOP) phenotypes are caused by mutations in the bone morphogenetic protein (BMP) type I receptor ACVR1. Hum Mutat.

[6] Pignolo RJ, Bedford-Gay C, Liljesthrom M (2016). The natural history of flare-ups in fibrodysplasia ossificans progressiva (FOP): a comprehensive global assessment. J Bone Miner Res.

[7] Pignolo RJ, Shore EM, Kaplan FS (2013). Fibrodysplasia ossificans progressiva: diagnosis, management, and therapeutic horizons. Pediatr Endocrinol Rev.

[8] Kaplan FS, Xu M, Glaser DL (2008). Early diagnosis of fibrodysplasia ossificans progressiva. Pediatrics.

[9] Kaplan FS, Chakkalakal SA, Shore EM (2012). Fibrodysplasia ossificans progressiva: mechanisms and models of skeletal metamorphosis. Dis Model Mech.

[10] Kaplan FS, Al Mukaddam M, Baujat G (2019). The medical management of fibrodysplasia ossificans progressiva: current treatment considerations. Proc Intl Clin Council FOP.

[11] Miao J, Zhang C, Wu S, Peng Z, Tania M (2012). Genetic abnormalities in fibrodysplasia ossificans progressiva. Genes Genet Syst.

[12] Shore EM, Xu M, Feldman GJ (2006). A recurrent mutation in the BMP type I receptor ACVR1 causes inherited and sporadic fibrodysplasia ossificans progressiva. Nat Genet.

[13] Di Rocco M, Baujat G, Bertamino M (2017). International physician survey on management of FOP: a modified Delphi study. Orphanet J Rare Dis.

[14] Chaikuad A, Alfano I, Kerr G (2012). Structure of the bone morphogenetic protein receptor ALK2 and implications for fibrodysplasia ossificans progressiva. J Biol Chem.

[15] van Dinther M, Visser N, de Gorter DJ (2010). ALK2 R206H mutation linked to fibrodysplasia ossificans progressiva confers constitutive activity to the BMP type I receptor and sensitizes mesenchymal cells to BMP-induced osteoblast differentiation and bone formation. J Bone Miner Res.

[16] Rahman MS, Akhtar N, Jamil HM, Banik RS, Asaduzzaman SM (2015). TGF-beta/BMP signaling and other molecular events: regulation of osteoblastogenesis and bone formation. Bone Res.

[17] Kaplan FS, Shen Q, Lounev V (2008). Skeletal metamorphosis in fibrodysplasia ossificans progressiva (FOP). J Bone Miner Metab.

[18] Wang RN, Green J, Wang Z (2014). Bone morphogenetic protein (BMP) signaling in development and human diseases. Genes Dis.

[19] Alessi Wolken DM, Idone V, Hatsell SJ, Yu PB, Economides AN (2018). The obligatory role of activin A in the formation of heterotopic bone in fibrodysplasia ossificans progressiva. Bone.

[20] Wang H, Behrens EM, Pignolo RJ, Kaplan FS (2018). ECSIT links TLR and BMP signaling in FOP connective tissue progenitor cells. Bone.

[21] Lees-Shepard JB, Yamamoto M, Biswas AA (2018). Activin-dependent signaling in fibro/adipogenic progenitors causes fibrodysplasia ossificans progressiva. Nat Commun.

[22] Kaplan FS, Al Mukaddam M, Pignolo RJ (2017). A cumulative analogue joint involvement scale (CAJIS) for fibrodysplasia ossificans progressiva (FOP). Bone.

[23] Towler OW, Shore EM, Kaplan FS (2020). Skeletal malformations and developmental arthropathy in individuals who have fibrodysplasia ossificans progressiva. Bone.

[24] Baujat G, Choquet R, Bouee S (2017). Prevalence of fibrodysplasia ossificans progressiva (FOP) in France: an estimate based on a record linkage of two national databases. Orphanet J Rare Dis.

[25] Shore EM, Feldman GJ, Xu M, Kaplan FS (2005). The genetics of fibrodysplasia ossificans progressiva. Clin Rev Bone Mineral Metab.

[26] Liljesthröm M, Pignolo RJ, Kaplan FS (2020). Epidemiology of the global fibrodysplasia ossificans progressiva (FOP) community. J Rare Dis Res Treat.

[27] Connor JM, Evans DA (1982). Genetic aspects of fibrodysplasia ossificans progressiva. J Med Genet.

[28] Mantick N, Bachman E, Baujat G (2018). The FOP Connection Registry: design of an international patient-sponsored registry for Fibrodysplasia Ossificans Progressiva. Bone.

[29] United States Census Bureau. U.S. and world population clock. United States Census Bureau website. https://www.census.gov/popclock/. Updated 2020. Accessed August 26, 2020.

[30] Sherman LA, Cheung K, De Cunto C, Kile S, Pignolo RJ, Kaplan FS. The diagnostic journey in fibrodysplasia ossificans progressiva: insights from the FOP registry. Presented at: ASBMR 2020 Virtual Annual Meeting, September 11–15, 2020.

